# Report of the Diseases of Edinburgh, for January, 1808

**Published:** 1808-03

**Authors:** J. Roberton

**Affiliations:** Surgeon


*4, St. James's Street.*

---

### *Report of the Diseases of Edinburgh, for January,* 1808. *By* Mr. J. Roberton, *Surgeon.*

THE mildness of the weather during the first ten or twelve days of this month, has seldom in any former year been surpassed in this country at the same period of the season. About the end of the first third of the month, however, the weather became very coarse, and there was, for a few days, considerable falls of rain with hard gales of wind. Nearer the end of the month the same unsettled state of weather, which has lately been so very common with us, seemed again likely to prevail. The thermometer, sometimes in the morning, stood at 8 or 9 degrees below the freezing point, and before night of the same day the weather was perfectly soft. About the end of the month changes of another kind took place; the weather became extremely tempestuous, attended with rain and snow in considerable quantities. And, on the evening of the 28th, as remarkable a state of weather existed, as perhaps ever was known in any country; for there seemed to be frost, rain, and snow alternating with each other every half hour, or they may almost be said to be co-existent. After this frost seemed to prevail, and with it the month terminated. For the most part of the month the barometer has been rather low.

Most of the diseases of last month still continue to exist, although many of them have become rather mild. Those which still seem to prevail with considerable violence are inflammatory diseases in general, particularly inflammation of the bowels among children. Among them, indeed, the diseases diseases which have lately proved most fatal have in general existed. In a variety of instances, a rapid succession of different diseases have affected them, such as chincough, succeeded by inflammatory complaints, and these again by febrile affections; and lastly by measles, which, although perhaps neither of them were individually violent, have in combination at last ended in the death of the patients. Independent of the most active treatment that could be employed, several of these complaints have been unaffected by it, and the patients have, as might be expected, died. Where examination after death was permitted, most astonishing quantities of black hardened fæces have been found in the intestinal canal, although, during the disease, proper means were used to prevent such accumulation, and apparently with pretty good effect.

Some of those diseases which had prevailed last month, or even sometime previous to it, and were beginning to abate, such as Cynanche Tonsilaris, seem to have increased in frequency.

Measles still continue to rage with unabated violence; indeed I believe, that the present epidemic has been more general in this place and its vicinity, than ever happened within the remembrance of any medical man at present living; and I am sorry to say it has been very fatal. The bowel complaint, which I formerly mentioned, seems very common among children affected with measles. There is likewise a febrile complaint, which often makes its attack about the desquamation of the eruption. In some instances it continues afterward at intervals to distress the patient for a week or two, and seems very destructive to those affected with such complaints. From the time of the year in which this disease has appeared, and the variety of obstinate symptoms and concomitant affections which do not usually accompany it, I believe its fatality has been very great among every description of people.

So convinced am I of the propriety of preserving in this disease the temperature of the room in which the patient is laid at about 60, or even lower, of Fahrenheit's scale, that I am in the habit of ordering a thermometer to be placed within the curtains of the bed, and as near the patient as possible, in order that the temperature may be regulated as near this point as possible. By this precaution, together with those rules which I laid down in my last Report, I have experienced the greatest success in practice relative to this complaint. In that Report, when speaking of the treatment, I mentioned, that blood taken from the temples by the application of leeches, was always useful, and often attended with most strikingly beneficial effects. Since I made that observation I have had further corroboration of the propriety of this practice. Its first effects seem almost immediately to be the alleviation of some of the more troublesome sympt oms that may be prevented; and I have seldom observed that those who were treated in that way were much distressed by the diarrhœa, &c. about which I likewise made some remarks, and which has always proved so difficult of removal. There was one family in particular where this practice appeared in the most striking point of vie w It consisted of five children, who had all suffered very recently from the chin-cough, and who were all about the same time affected with the measles, which were in each of them seemingly of similar severity. About the beginning of the eruption of measles, in this family, in order to remove what is vulgarly known by people in this part of the world by the name of the chin-cough, leeches were applied by the parents to the side of the head in two of the children, which bled very profusely; but to the other three, no such application was made. The disease continued the usual time, and gradually abated in these two without leaving any troublesome symptoms; but before this application was made to the other three, several bad symyptoms supervened, and independently of the most active treatment that could afterwards be applied, they all died. The two that were bled have had no diarrhœa, nor did the cough trouble them, except during a very few days after the eruption had disappeared.

I have seen several cases of Erysipelas, both on the face and on other parts of the body. The inflammatory symptoms, as indicated by the pulse, did not in any of them run very high. I have not been informed of any cases of this disease during the present month proving fatal.

By the bye, I may mention, that it appears to me, that sufficient attention has never been paid to the sequela of this disease, which is often productive of much distress to the patient.

After the violence of the systematic affection, in general originally causing this disease, has subsided, or, what is nearly the same in its consequences, the erythema of Cullen, where the systematic affection is wholly in consequence of the local inflammation, many instances of both these affections terminate in sores of the most obstinate and disagreeable kind, generally upon those parts of the body body where the diseases had appeared externally. Independently of every other consideration, the great extent of surface which these sores usually occupy, render them very distressing. The legs, which are commonly the seat of these complaints, are often entirely affected by them, rendering the ill-fated patient completely incapable either of occupation or amusement. Such sores often continue for many years, and most frequently during the remainder of the patient's life. The parts affected by such diseases assume a livid colour, discharging ill-conditioned matter from various parts of the diseased surface. In time, longer or shorter, according to the habit of body of the patient, extensive ulcerations appear on the parts, causing most acute pain, and discharging immense quantities of variously coloured matter. And, from time to time, hæmorrhage, to a great extent occurs, and debilitates the patient very much. To these I may add the immense swellings that almost always affect the limbs.

The applications recommended for the cure of such affections, have indeed been of very little service. Ointments have only been useful in keeping the parts soft and somewhat easy. I have in some instances succeeded in the complete cure of slight cases by bandaging, and the use of an ointment, now completely abolised from modern Pharmacopeias, and of course only to be found in very old books of that description, known by the name of unguentum nutritum *; but in cases of long standing and great obstinacy, I have used this application for years, without deriving any material advantage from it.

Since I have so successfully used the Cantharides in sores of different kinds †, among which I include the affection I have alluded to above, I have, except the properly regulated use of bandages, laid aside every other external application. I may indeed make another exception to this rule, but it is only an occasional one; namely, the application of caustic substances to such parts, particularly the edges of old ulcers, as may have acquired a degree of hardness, almost of the consistence of cartilage. In some of these cases, I have even been obliged to separate these hard portions with the knife. There is a peculiarity of

---

* It is composed of ℥j. of litharge, ℥ij. of vinegar, and ℥iij. of Florence oil, mixed in the order I have mentioned them.

† See Practical Treatise on the Internal Use of Cantharides in gleet, leucorrhœa, and obstinate sores.

action action too, which sometimes exists to a considerable extent on diseased surfaces, and which internal medicines, however long their use may be continued, can never be overcome. But when the system has been sufficiently excited by the internal use of cantharides, and the vessels which caused the morbid action destroyed by the application of a blister to the part, I have never failed in the complete removal of such complaints.

In one instance, a very curious circumstance occurred to me, while using the cantharides in a case of the above description, which had continued to affect the right leg of a patient for several years. After he had continued to use this medicine for ten or twelve days, and the sores of the leg had become considerably inflamed, he was seized with all the symptoms of erysipelas, and that affection spread to a greater extent than it had originally done. In a few days, however, it subsided, and by the application of bandages and pads of linen cloth alone, the original affection entirely disappeared in a few weeks.

From the resemblance in many instances of the Cynanche Tonsillaris, lately so very prevalent among us, combined with the eruptive stage of the measles, to scarlatina anginosa, I thought I was warranted to state in my last Report, that those Gentlemen were possibly in some mistake, who thought they had observed scarlatina anginosa about the beginning of last month, particularly as the cases said to have been met with were few in number, and because the epidemic's disappearance was likewise said to be so very rapid. About the end of last, and since the beginning of this month, however, this disease has more decidedly assumed those appearances which characterize it. This induces me to believe, that the opinion of its existence about the beginning of last month was not without foundation. Still, however, in many instances, to discriminate accurately between this disease and the measles, combined with cynanche, is very difficult. Indeed so exact a resemblance on the present occasion do they seem to bear to each other that I have lately seen several cases of scarlatina, which could only be distinguished from the other complaint, by the patient assuring me that they had previously been affected by the measles.

I have neither seen nor heard of any case of great severity. Many of them yield completely in a few days, by the use of a gargle, made of the decoction of Peruvian bark, acidulated with nitric acid; and if to this be added the internal use of the bark, even in severer cases, a complete plete plete cure is obtained in a few days. I have only in one case found it necessary to apply a blister to the throat.

When I consider the number of accidents, that during this month occur to skaiters and others, in this as well as in every other part of our island, from the falling in of ice, it appears to me very strange, that no plan, with which I am acquainted, has ever been adopted, to give the drowning person a chance of escape. In the neighbourhood of this city, where skaiting is a very favourite amusement, there is seldom a season that one or more persons are not drowned in the lakes or *lochs*, as we call them. Yet this very necessary consideration seems entirely to have escaped the notice of those to whom we ought to look for improvements of this kind.

It is allowed by every one, that most of the persons who fall victims in this way, do so from want of something to catch hold of after they have fallen into the water. They are therefore under the necessity of endeavouring to lay hold of the edge of the broken ice, which almost always gives way, and the unfortunate victim is plunged under it, from which he is seldom able to extricate himself, and therefore must perish. His companions too, in stretching out their arms, or in holding long branches of trees to assist the escape of the drowning person, often share the same fate.

It occurs to me, that almost all these unfortunate circumstances may be prevented, by proper attention being paid to them. If poles, iron rings, or such fixed points were placed at various parts of the lakes, and ropes or chains of sufficient length attached to them long enough to reach any part of these lakes, by the assistance of those on the spot at the time, there might always be afforded a considerable chance of escape for the drowning person.

